# Online Monitoring of Transformer Winding Faults Based on Pulse Coupling Injection

**DOI:** 10.3390/s26092914

**Published:** 2026-05-06

**Authors:** Zetong Wang, Yuhan Zou, Junhao Ma, Zongnan Liu, Xinyu Peng, Tianran Zhang, Sizhe Xiang, Chenguo Yao, Shoulong Dong

**Affiliations:** State Key Laboratory of Power Transmission Equipment Technology, Chongqing University, Chongqing 400044, China; 202411131264@stu.cqu.edu.cn (Z.W.); 202411021151t@stu.cqu.edu.cn (Y.Z.); 202311021093t@stu.cqu.edu.cn (J.M.); 202411021047@stu.cqu.edu.cn (Z.L.); 202411131115t@stu.cqu.edu.cn (X.P.); 202411131260@stu.cqu.edu.cn (T.Z.); 202211021122t@stu.cqu.edu.cn (S.X.); yaochengguo@cqu.edu.cn (C.Y.)

**Keywords:** transformer winding fault, pulse frequency response method, capacitive coupling sensor, live monitoring, MSCNN–Transformer–PGA model

## Abstract

**Highlights:**

**What are the main findings?**

- Development of a modular semi-ring capacitive coupling sensor with a spring-loaded self-adaptive mechanism and multi-DOF injection interface, enabling non-contact, bushing-size-independent live monitoring.
- The proposed MSCNN–Transformer–PGA deep learning model achieves a high fault classification accuracy of 97.6%, relying solely on impulse frequency response data.

**What are the implications of the main findings?**

- These findings effectively overcome the technical bottleneck of traditional offline detection, enabling safe and real-time live monitoring of transformers.
- This hardware–software collaborative approach demonstrates strong robustness against complex electromagnetic noise, providing a highly reliable predictive maintenance solution for smart grids.

**Abstract:**

Aiming at the problems with traditional transformer winding deformation detection, requiring power outages, low signal-to-noise ratios for online monitoring, and insufficient feature extraction, this paper proposes a live monitoring and intelligent diagnosis method based on pulse-coupled injection. At the hardware level, a semi-ring capacitive coupling sensor is developed and designed, which realizes non-contact injection of high-frequency pulse signals and high-SNR extraction without a power outage. The reliability of the system under complex working conditions is verified by field experiments on multiple actual 110 kV transformers. At the algorithm level, an innovative MSCNN–Transformer–PGA deep composite model fused with prior electromagnetic physical knowledge is constructed and combined with the transformer equivalent circuit model. The model uses a multi-scale convolution to extract local details of frequency response signals, adopts Transformer to establish the global sequence dependence, and introduces a Physics-Guided Attention mechanism (PGA) to adaptively focus on the key fault physical frequency bands. The experimental results show that the proposed method effectively overcomes electromagnetic noise interference, and the fault classification accuracy of single-modal pulse frequency response data reaches 97.6%, providing a high-precision online monitoring solution for the safe operation and maintenance of transformers.

## 1. Introduction

Against the background of the continuous growth in global energy demand, power systems have become the core infrastructure supporting the operation of the economy and society, and their safe and stable operation is directly related to the development of national economies and the protection of peoples’ livelihoods [1,2]. As a key piece of equipment for energy conversion and transmission in power systems, power transformers undertake core functions, such as voltage level regulation and efficient power distribution, and the reliability of their operating status directly determines the power supply quality and safety level of a power system [3]. However, during long-term service, transformers are faced with multiple complex working conditions, such as short-circuit current impacts, electromagnetic force action, and temperature cycle changes, which are prone to various faults. Among them, mechanical faults are a primary cause of unplanned transformer outages. According to condition observations and statistics from the International Council on Large Electric Systems (CIGRE), winding mechanical deformation and displacement account for approximately 15% to 20% of all major power transformer failures worldwide. The huge electromagnetic force generated by short-circuit currents often causes cumulative and irreversible deformation of windings. If not detected in a timely manner, these minor mechanical displacements progressively develop into serious faults, such as inter-turn short circuits and insulation breakdown, leading to equipment damage and large-scale power outages. Therefore, the winding deformation monitoring system proposed in this study is particularly suitable for large-scale power transformers in continuous operation at critical grid nodes, as well as for aging transformers with a history of short-circuit impacts. For these critical assets, traditional offline testing is highly impractical due to the prohibitive downtime costs, making live monitoring essential for capturing latent defects and enabling predictive maintenance. Therefore, carrying out research on accurate and efficient monitoring technology for transformer winding faults is of crucial practical significance for ensuring the safe and stable operation of power systems [4,5].

With the far-reaching advancements in intelligence and the digital transformation of power systems, fault detection technology is rapidly developing in online, real-time and intelligent directions. Frequency response analysis (FRA), with its significant advantages of accuracy, economy and non-destructiveness, has become the industry-recognized most effective method for transformer winding deformation detection and is widely used in transformer condition assessments. Based on the characteristic that a transformer winding can be considered equivalent to a passive two-port network composed of linear resistors, inductors and capacitors under high-frequency excitation, this method judges the mechanical state of the winding by analyzing the changes in the winding frequency response curve. However, traditional FRA technology needs to carry out tests when a transformer is out of power, which conflicts with the core demand of modern power systems of uninterrupted power supply. Frequent power outages not only affect the continuity of power supply, but also increase operation and maintenance costs, making it difficult to meet the actual demands of lean operation and maintenance of the power system. Therefore, developing online FRA detection technology without power outages to realize live monitoring of winding deformation state has become a key technical problem that urgently needs to be solved in the field of power equipment condition monitoring [6,7,8,9].

At present, scholars at home and abroad have carried out numerous explorations of online detection technology for transformer winding faults, forming various detection schemes, such as the short-circuit impedance method, low-voltage pulse method, and sweep frequency impedance method [10,11,12,13]. Although the short-circuit impedance method is simple to operate and has clear physical significance, its sensitivity is limited, making it difficult to detect early minor faults. The low-voltage pulse method has high sensitivity but weak anti-electromagnetic interference ability, which makes it difficult to interpret the data in complex electromagnetic environments. It has only been a short time since the sweep frequency impedance method’s advent, and no mature industry standards have been formed yet. In explorations of the online application of frequency response analysis, impulse frequency response analysis (IFRA) has shown good development potential due to its characteristics of short detection time and adaptability to online application scenarios. However, some studies use natural transient signals as excitation sources, which have problems such as uncontrollable amplitude and insufficient frequency bandwidth. Other studies have attempted to inject high-frequency signals through the bushing end screen or neutral point, but they are faced with technical bottlenecks such as weak response signals easily masked by noise, sensor installation affecting equipment insulation safety, and injection loop parameters interfering with detection results, and no mature engineering application schemes have been formed yet [14,15,16,17].

Aiming at the problems existing in current online detection technologies, such as poor controllability of excitation signals, low signal-to-noise ratios, insufficient equipment compatibility, and limited fault identification accuracy, this study proposes a live monitoring technology for transformer winding faults based on pulse coupling injection. By integrating confirmatory experimental tests with field installation and application research, a complete online monitoring system demonstrating high safety, reliability and accuracy is constructed. Through the customized design of a high-voltage pulse generator and a capacitive coupling sensor, this technology facilitates the controllable injection of excitation signals and the real-time acquisition of frequency response data without affecting the normal operation of transformers or changing the original wiring mode of power systems. Meanwhile, at the intelligent diagnosis level, this study innovatively constructs an MSCNN–Transformer–PGA deep hybrid diagnostic model incorporating electromagnetic physical prior knowledge. The model uses a Multi-Scale Convolutional Neural Network (MSCNN) to extract the multi-band local details of IFRA signals, establishes global sequence dependencies combined with the Transformer encoder, and adaptively focuses on key physical frequency bands through the PGA mechanism [18,19,20,21,22]. Combined with equivalent circuit model analysis, the proposed method effectively suppresses the interference of complex electromagnetic noise and achieves high-precision classification of fault types utilizing impulse frequency response data. This paper presents a hardware–software collaborative framework for transformer online monitoring. Specifically, the hardware system, centered on a customized capacitive coupling sensor, provides the physical capability for non-invasive pulse injection and data acquisition. This is seamlessly integrated with the software component—a physics-guided deep learning model—which performs intelligent fault classification. The effectiveness of this collaborative approach is validated through both simulations and field case studies. This research is expected to addresses the limitations of traditional detection technologies, providing a non-outage, high-precision and intelligent monitoring solution for transformer winding faults, thus contributing to the construction of more reliable and efficient modern power systems.

## 2. Principle of Live Monitoring Method for Winding Faults and Sensor Design

### 2.1. Principle of Impulse Frequency Response Analysis

**Figure 1:**
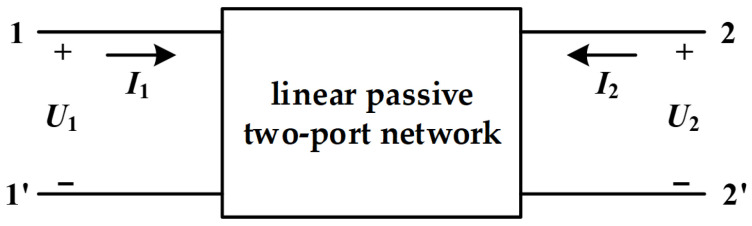
Equivalent circuit model of transformer two-port network.

The IFRA method builds upon the basic principles of FRA. Its core principle involves applying a nanosecond pulse signal to a transformer winding, measuring its subsequent response signal, and processing this data to extract the winding’s response characteristics across different frequencies. The transformer winding can be modeled as a passive two-port network, as illustrated in Figure 1.

While the ferromagnetic core introduces nonlinear effects such as hysteresis and saturation, the transformer is conventionally treated as a linear passive two-port network for FRA/IFRA applications. This simplification is justified because mechanical deformations primarily affect the high-frequency response, where the core’s magnetizing influence is minimal and linear stray parameters dominate.

Upon applying an excitation voltage signal *u*(*t*) to this equivalent circuit, its governing differential equation can be derived according to Kirchhoff’s laws, as expressed in Equation (1).

(1) $$u(t)=R_{i}(t)+L\frac{di(t)}{dt}+\frac{1}{C}\int_{0}^{t}i\left(\tau \right)d\tau$$

The IFRA method employs a nanosecond pulse signal as the excitation source. The time-domain expression of this nanosecond pulse signal *u_p_*(*t*) is determined by the specific pulse type. For instance, the expression for a standard rectangular pulse is given by Equation (2).

(2) $$u_{p}(t)=\left\{\begin{array}{l} A,0\leq t\leq T_{P}\\ 0,t>T_{P} \end{array}\right.$$

where *A* is the pulse amplitude and *T_p_* is the nanosecond pulse width.

A rectangular nanosecond pulse is specifically selected as the excitation source because its Fourier transform exhibits a continuous and exceptionally broad frequency spectrum. According to signal processing theory, a single pulse with a nanosecond-level rise time can simultaneously excite the passive components of a transformer winding across a broad frequency band. This broad spectral characteristic forms the theoretical foundation of IFRA, enabling rapid, single-shot frequency response acquisition. Compared to the time-consuming point-by-point frequency sweeping employed in traditional SFRA, this rectangular impulse is highly suited for transient extraction and real-time online monitoring.

Upon applying this nanosecond pulse signal to the input terminal of the equivalent circuit, the output response current *i_p_*(*t*) is derived, according to the convolution theorem, by convolving the input pulse signal *u_p_*(*t*) with the circuit response *h*(*t*), as expressed in Equation (3).

(3) $$i_{p}(t)=u_{p}(t)\cdot h(t)=\int_{-\infty }^{+\infty }u_{p}(\tau )h(t-\tau )d\tau$$

By measuring the excitation and response signals, the winding’s response characteristics across different frequencies can be computed. Comparing this calculated frequency response curve against a healthy baseline enables the detection of faults, such as winding deformation. When such deformation occurs, parameters such as inductance, capacitance and other elements within the winding’s equivalent circuit change, which in turn induces variations in the frequency response curve, including resonant frequency shifts and amplitude fluctuations.

### 2.2. Design of Capacitive Coupling Sensor

A bushing capacitive coupling sensor is a critical component of IFRA-based winding detection devices. Its core function is to facilitate the injection of pulse signals via capacitive coupling, thereby enabling the diagnosis of winding faults and the evaluation of equipment health. Although traditional fully enclosed sensors partially meet the insulation and conductivity requirements of transformers, they present significant limitations in practical applications, including complex installation and difficult maintenance. To address these challenges, a novel sensor is proposed in this study. Featuring a semi-ring design, the sensor undergoes rigorous structural optimization and electrical parameter tuning to meet the demands of online monitoring in high-voltage environments, demonstrating high sensitivity and reliability. Structurally aligning with the physical characteristics of transformer high-voltage bushings, this sensor employs a non-intrusive design to facilitate the injection and measurement of excitation signals.

**Figure 2:**
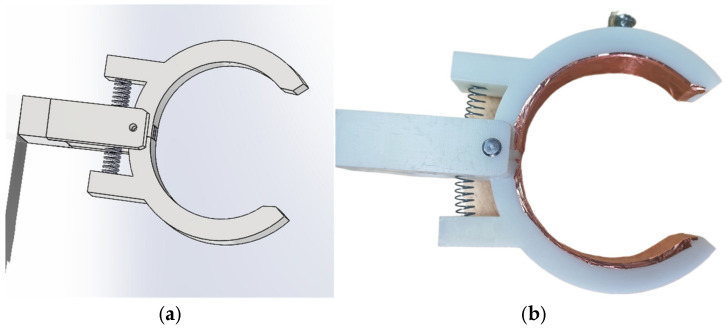
Structure diagram of capacitive coupling sensor. (**a**) Model diagram; (**b**) Physical diagram.

This sensor consists of a thin metal strip, an insulating layer, and internal insulating oil, as illustrated in Figure 2. Exhibiting high electrical conductivity and mechanical strength, the metal strip effectively captures the operating state of the bushing, transmits induced signals, and provides reliable data for the monitoring equipment. The insulating layer and insulating oil provide critical dielectric strength, ensuring the sensor’s safety and operational stability in high-voltage environments.

**Figure 3:**
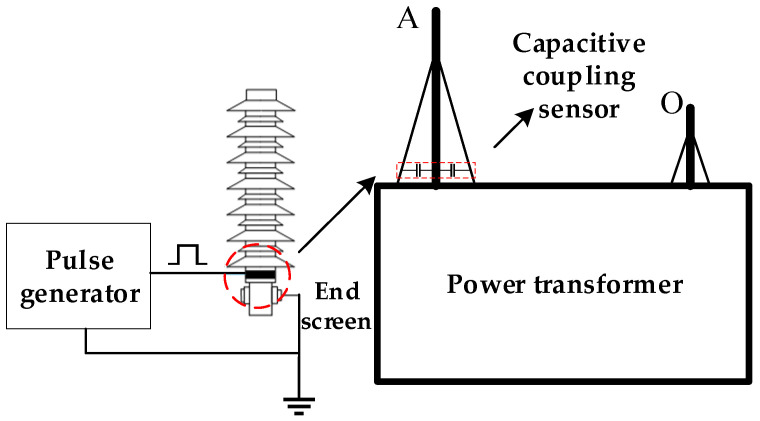
Schematic diagram of injection principle and installation position of capacitive coupling sensor. (A: Phase A; O: neutral point).

The capacitive coupling sensor is secured beneath the lowermost shed of a transformer bushing. Comprising the thin metal strip, the bushing’s inner guide rod, and the internal insulating medium, its structure forms a stable capacitive unit resembling a coaxial configuration, as illustrated in Figure 3. This design facilitates effective signal coupling and extraction while enhancing the reliability and safety of the measurement process. Mechanically secured by an insulating rod, the sensor enables rapid installation and removal. This insulating rod exhibits both high dielectric strength and sufficient mechanical robustness, maintaining the sensor’s stability during both installation and operation. In contrast, traditional fully enclosed sensors typically require long-term mounting on transformers and are difficult to disassemble once installed, introducing significant inconvenience during equipment maintenance and replacement. The rapid deployment capability of this novel sensor allows maintenance personnel to monitor and maintain transformer windings more effectively, thereby improving overall operational efficiency.

This capacitive coupling sensor can perform measurements without requiring transformer outages, whereas traditional fully enclosed sensors are incapable of online detection under live conditions due to their structural and installation limitations. This installation advantage of the semi-ring capacitive sensor enhances the flexibility and efficiency of detection, enabling real-time monitoring of the bushing’s operating status without disrupting normal transformer operations. Furthermore, to reduce energy loss and improve signal transmission efficiency, impedance matching is optimized between the sensor, the external signal source, and the receiving circuit.

**Figure 4:**
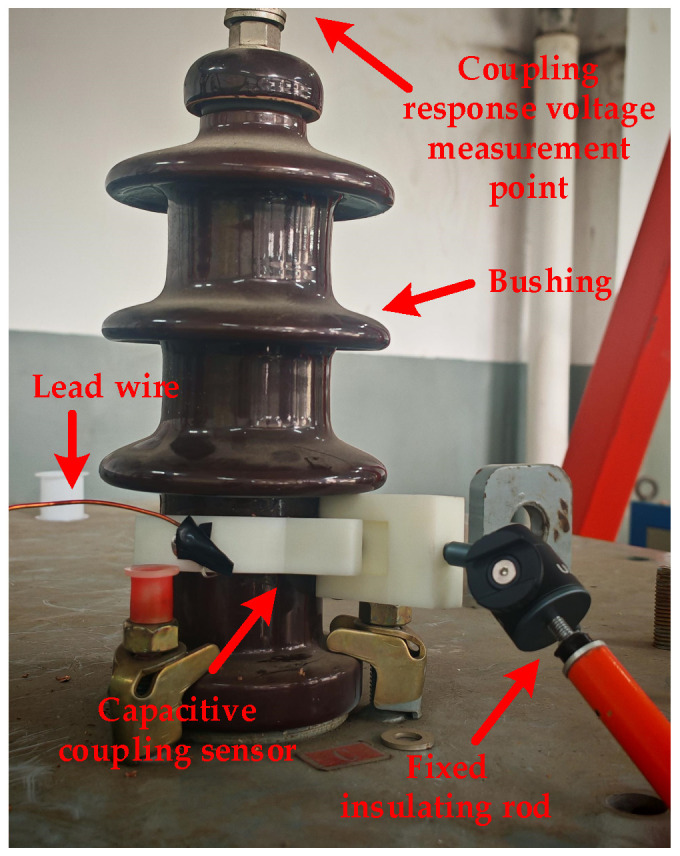
Site of bushing coupling injection test.

To verify that the coupling ratio of the designed sensor meets design requirements, an offline coupling injection test is conducted on a capacitive transformer bushing in this study. The capacitive coupling sensor featuring the semi-ring design is mounted around the root of the bushing under test. A pulse voltage excitation is applied to the sensor, and the coupled response voltage signal is measured at the inner guide rod of the bushing. The test site layout is illustrated in Figure 4.

**Figure 5:**
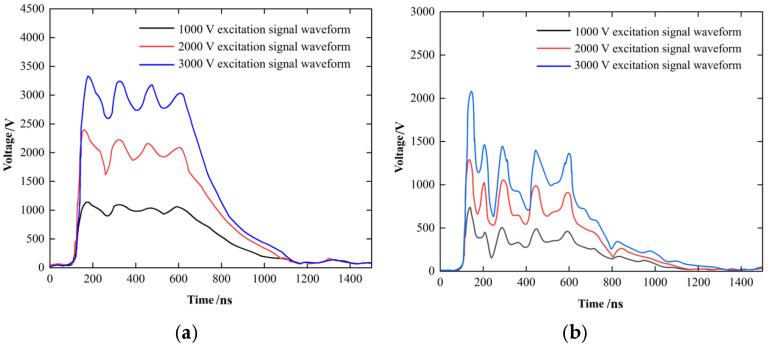
Excitation signal and response signal of bushing coupling injection. (**a**) Excitation voltage signal; (**b**) Coupling response voltage signal.

Experimental results validate the effectiveness of the semi-ring capacitive coupling sensor design, as illustrated in Figure 5. Figure 5a displays the excitation signal, and the coupled response voltage depicted in Figure 5b exhibits a high correlation with this excitation source. The energy transfer efficiency of the main frequency component achieves 40% of the design target, and background noise is effectively suppressed, confirming the sensor’s high signal-to-noise ratio characteristics in complex electromagnetic environments.

Compared to traditional fully enclosed structures, the semi-ring design optimizes electric field distribution and maintains comparable signal coupling efficiency within the key detection frequency band, while offering three distinct advantages: first, the open structure enables non-contact installation, avoiding the power outages and bushing disassembly required by traditional sensors; second, the modular design allows the sensor to accommodate transformer bushings of different voltage levels; and third, the dual-shield architecture preserves signal transmission quality and reduces partial discharge risks by 70%. By overcoming the limitations of traditional sensors, this novel design not only improves detection efficiency and safety but also provides a more convenient and reliable solution for transformer maintenance and management.

## 3. Winding Fault Simulation and Field Installation Application

In the previous chapter, a high-sensitivity capacitive coupling sensor was designed and verified, establishing a hardware foundation for pulse injection and data acquisition. To elucidate the specific influence mechanisms of typical faults on the frequency response characteristics of windings, this chapter first establishes an equivalent circuit model of a transformer winding for simulation analysis. Subsequently, this hardware system is deployed for field testing on actual transformers.

### 3.1. Transformer Winding Fault Simulation

#### 3.1.1. Equivalent Circuit Construction

An equivalent circuit model is constructed based on a trapezoidal network topology. Referring to the design parameters of the model transformer, its high-voltage winding consists of 30 wire cakes, consequently, the trapezoidal network contains 30 corresponding basic units.

Constructing this circuit involves a substantial number of components. Manually adding and connecting each component according to the trapezoidal network structure would entail an impractical workload. Therefore, leveraging the capabilities of MATLAB\Simulink (version R2025a), a script was developed to automatically instantiate thousands of components within Simulink and facilitate their orderly interconnection, thereby automating the circuit model construction process.

**Figure 6:**
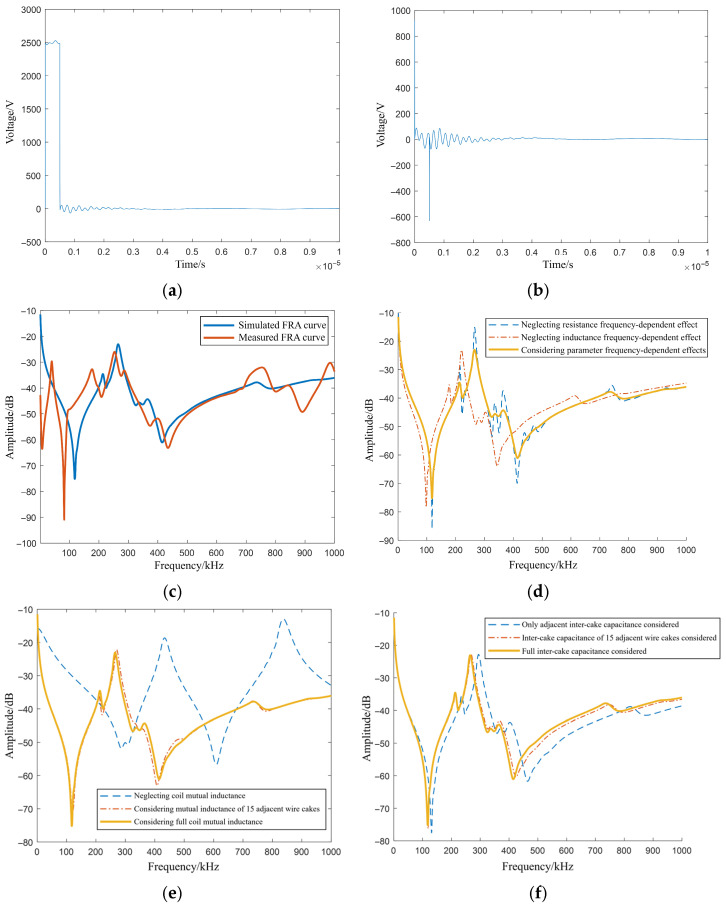
Simulation time-domain results and simulated FRA curves. (**a**) Excitation signal; (**b**) Response signal; (**c**) Simulated frequency response curve; (**d**) Frequency response comparison without considering parameter frequency-dependent effects; (**e**) Frequency response comparison without considering distant mutual inductance; (**f**) Frequency response comparison without considering distant inter-cake capacitance.

Based on the definition of frequency response analysis, the frequency response of the simulated equivalent circuit is analyzed. A pulse voltage excitation with a pulse width of 500 ns and an amplitude of 5 kV is applied to the equivalent circuit model, with the excitation and response signals acquired simultaneously. The frequency response of the equivalent circuit is defined as the ratio of the Fourier transform of the response signal to that of the excitation signal. The excitation signal is illustrated in Figure 6a, and the corresponding response signal is depicted in Figure 6b. It is observed that under the load effect of the winding circuit, the excitation signal exhibits pronounced oscillations both during the pulse’s flat-top phase and following its termination, whereas the response signal generates sharp spikes at the transient edges. The transient waveform dissipates after approximately 10 μs. The resulting frequency response curve within the range of 1 kHz–1 MHz, calculated via the Fourier transform ratio of these two signals, is presented in Figure 6c. Furthermore, Figure 6d displays the FRA curve without considering the frequency-dependent effects of resistance and inductance.

A comparison between the simulated and measured FRA curves in Figure 6c reveals a similar basic trend and strong agreement. The resonance points in the low-frequency range of the measured FRA curve—with the exception of the first pair of resonance peaks and valleys—can be identified near the corresponding frequency points on the simulated curve. Furthermore, Figure 6d–f demonstrates that the curves obtained without considering frequency-dependent effects, distant mutual inductance, and distant inter-cake capacitance exhibit larger deviations from the measured curve. This indicates that incorporating these frequency-dependent parameters is beneficial for establishing a more accurate circuit model.

Compared to the measured FRA curve, the simulated curve lacks information regarding the first pair of resonance peaks and valleys. This discrepancy is likely attributable to the omission of the iron core effect. Investigations of numerous power transformer frequency responses within the power grid reveal that the initial pair of resonance peaks and valleys is often closely associated with the iron core. In the low-frequency range where these initial resonance features are located, although the frequency reaches the kHz level, the excitation effect of the iron core remains influential. Incorporating the equivalent magnetic circuit of the iron core in future work would further improve the model’s accuracy.

#### 3.1.2. Inter-Cake Short Circuit

**Figure 7:**
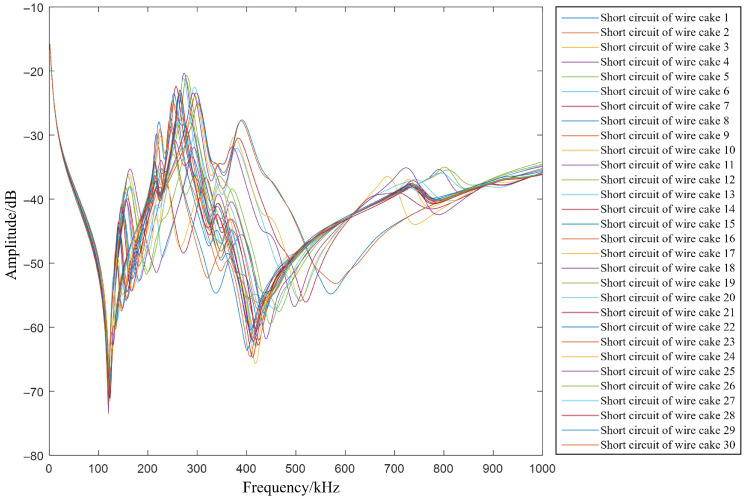
FRA curves under short-circuit faults at different positions.

Simulating an inter-cake short-circuit fault is straightforward, requiring only the connection of the two ends of the affected coils via a low-value resistor. Recognizing that contact resistance persists even in a metallic short circuit, this fault resistance is set to 0.1 Ω. To investigate the variation trends of the FRA curve when short-circuit faults occur at different locations, each wire cake is short-circuited sequentially. Figure 7 illustrates the corresponding FRA curves for various short-circuit locations.

As observed in Figure 7, following an inter-cake short circuit, the resonance points of the curve shift significantly, and those in the mid-frequency band tend to migrate toward the high-frequency region. Overall, the low-frequency band is relatively less affected, whereas the mid- and high-frequency bands exhibit pronounced variations. The measured frequency response data for inter-cake short-circuit faults also exhibit similar characteristics, which substantiates that the circuit model in this study corresponds well with the physical behavior of an actual transformer winding.

**Figure 8:**
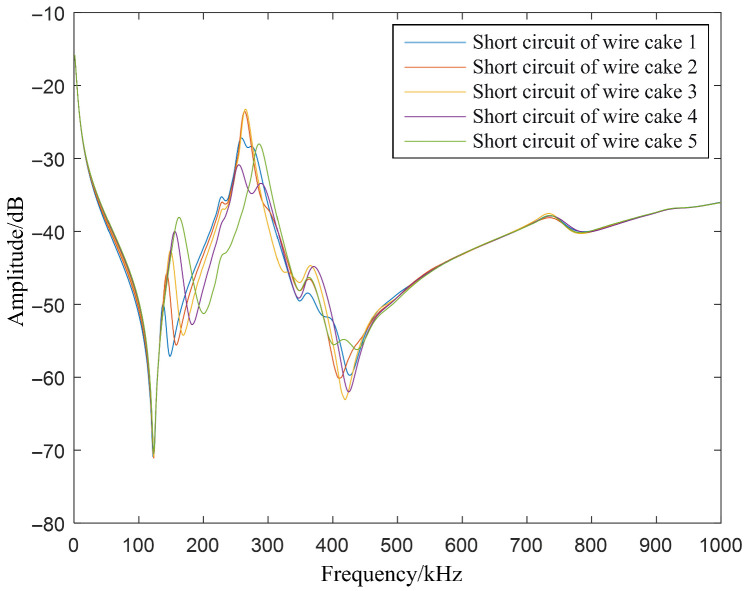
FRA curves under short-circuit conditions from the 1^st^ to the 5^th^ wire cake.

To illustrate this phenomenon more clearly, Figure 8 focuses on the comparison of FRA curves for short-circuit conditions across the first five wire cakes.

Following a short circuit, the first resonance valley near 120 kHz does not shift significantly, but a pair of pseudo-resonance points emerges subsequent to this valley. As the fault location progresses from the first to the fifth wire cake, these pseudo-resonance features shift toward the high-frequency region. Moreover, the lower the fault position, the more pronounced the curve deviation becomes. It is also observed that the resonance points not only shift toward higher frequencies but also migrate upward in amplitude. This behavior is attributed to a decrease in the circuit’s overall impedance following the short circuit, which leads to reduced signal attenuation across the system and, consequently, a higher signal transmission gain. In the amplitude spectrum, this manifests as an upward translation of the curve. The overall trend of the curve following the short circuit can be characterized as a shift toward higher frequency bands. Throughout these analyses, the fault-free (healthy) state serves as the baseline for evaluating fault evolution. Compared to this healthy baseline, an inter-cake short circuit induces a distinct high-frequency shift in the resonant peaks. As illustrated in Figure 7 and Figure 8, an increase in the number of shorted wire cakes results in a progressively more pronounced deviation from the initial healthy signatures, demonstrating a clear migration of the resonance features.

#### 3.1.3. Radial Deformation

**Figure 9:**
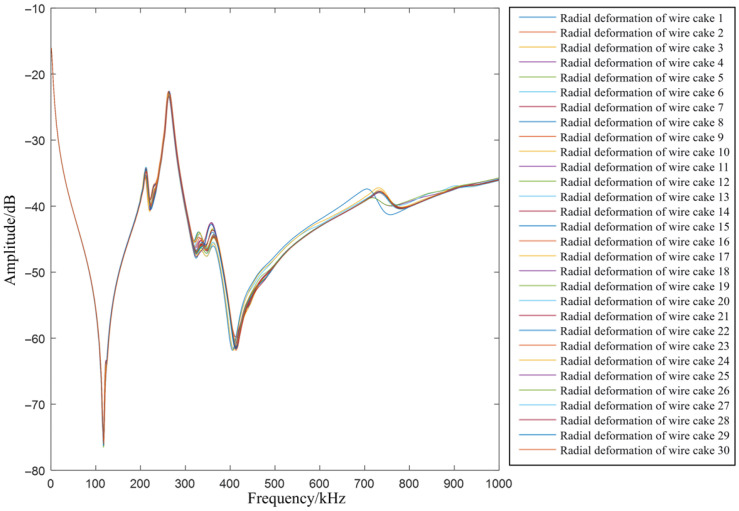
FRA curves under radial deformation at different positions.

Since high-voltage windings are generally positioned on the outer side, electromagnetic analysis indicates that the resultant forces are directed outward. Consequently, radial deformation in high-voltage windings typically manifests as an outward protrusion. According to finite element modeling and simulation, both the self-inductance and mutual inductance of the coils increase following such a protrusion. Therefore, in this section, the corresponding values within the inductance matrix are increased to simulate radial outward deformation. Figure 9 illustrates the FRA curves for radial deformations occurring at various locations.

As observed in the figure, the influence of radial deformation on the FRA curve is relatively subtle. The low-frequency band remains nearly stationary, while a slight shift occurs in the mid- and high-frequency regions; however, the overall trajectory of the curve remains unaltered. The measured FRA curves for radial deformation are consistent with these simulation results. Although the healthy baseline is not superimposed to maintain visual focus on fault severity increments, a comparison with the inherent normal condition reveals that radial deformation exerts a localized impact. Specifically, in the high-frequency range (>500 kHz), a detectable amplitude deviation from the healthy baseline is consistently observed, indicating that even minor structural changes result in a systematic shift in the resonant valleys relative to the healthy state.

#### 3.1.4. Variation in Inter-Cake Spacing

In practice, the fault of inter-cake spacing variation corresponds to the displacement of a specific wire cake, which alters the distance between that cake and its adjacent ones. When a wire cake moves downward, the distance to the subsequent cake decreases, resulting in an increase in the corresponding longitudinal capacitance. Concurrently, the distance to the preceding cake increases, leading to a decrease in the longitudinal capacitance on that side. Since this displacement is relative, an upward movement yields similar characteristics. In the fault simulation, the longitudinal capacitances on both sides of the target wire cake are adjusted in opposite directions to simulate vertical coil displacement—specifically, the fault of inter-cake spacing variation.

**Figure 10:**
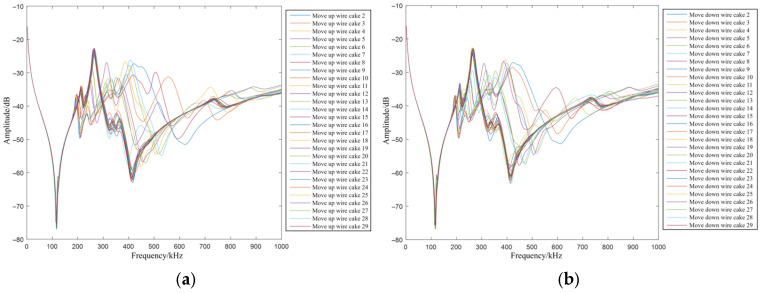
FRA curves under inter-cake spacing variation at different positions. (**a**) Upward movement of wire cakes; (**b**) Downward movement of wire cakes.

Figure 10 illustrates the FRA curves for the downward and upward movement of each wire cake. As observed in the figure, the low-frequency band remains nearly stationary, indicating that changes in capacitance primarily affect the mid- and high-frequency regions. Taking the healthy state as the inherent reference for the evolutionary process, the signatures presented in Figure 10 reflect the continuous migration of resonant points under inter-cake spacing variations. The results demonstrate that as the spacing deviates from its design value, the frequency response signatures undergo a consistent and measurable transformation, confirming the model’s ability to track the progression of mechanical displacement from its original healthy configuration.

**Figure 11:**
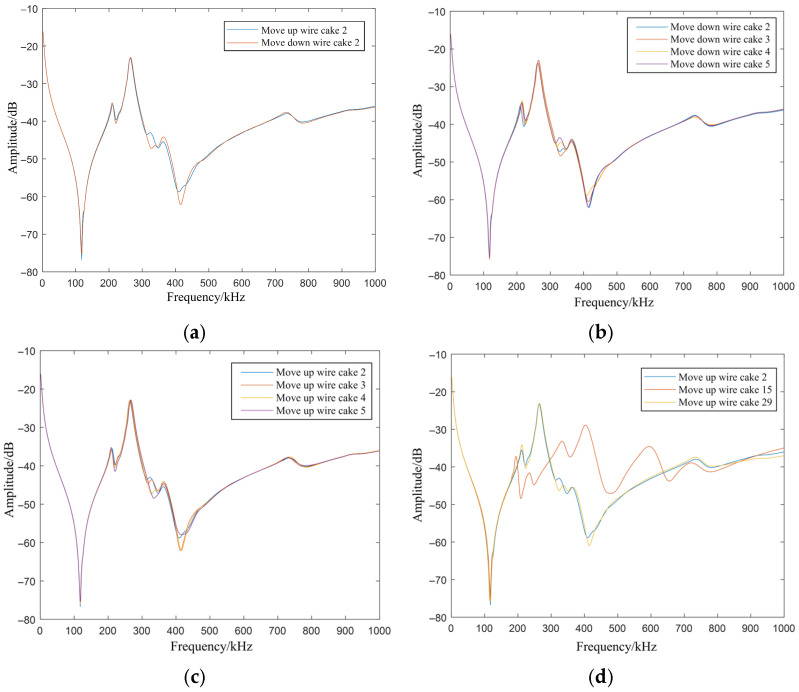
Comparison of FRA curves under inter-cake spacing variation in different disk cases. (**a**) FRA curves for different movement directions of the second disk coil. (**b**) FRA curves for downward movement of different disk coils. (**c**) FRA curves for upward movement of different disk coils. (**d**) Comparison of FRA curves for upward movement at symmetric and asymmetric positions.

Observing Figure 11: (a) Shows the influence of different movement directions of wire cake 2 on the FRA curves. The upward and downward movement of wire cake 2 only changes the longitudinal capacitance distribution between wire cake 2 and wire cakes 3 and 4, and the capacitance variation is as small as the nF level. This reflects that the curve in the 300–500 kHz range is relatively sensitive to longitudinal capacitance parameters. (b,c) Compare the FRA curves under inter-cake spacing variation faults at different positions. Almost no difference can be seen within 200 kHz, a slight shift occurs in the 200–300 kHz range, and various degrees of shift appear at 300–500 kHz. In (d), the FRA curves obtained under the upward movement of wire cake 2 and wire cake 29 are very similar, while both are obviously different from that of wire cake 15 upward movement. The relationship between the similarity of the curves and the spatial symmetry of the coil positions is consistent with that in the case of inter-cake short circuit, and will not be repeated here.

### 3.2. Field Installation and Application Experiment

#### 3.2.1. Experimental Procedure

**Figure 12:**
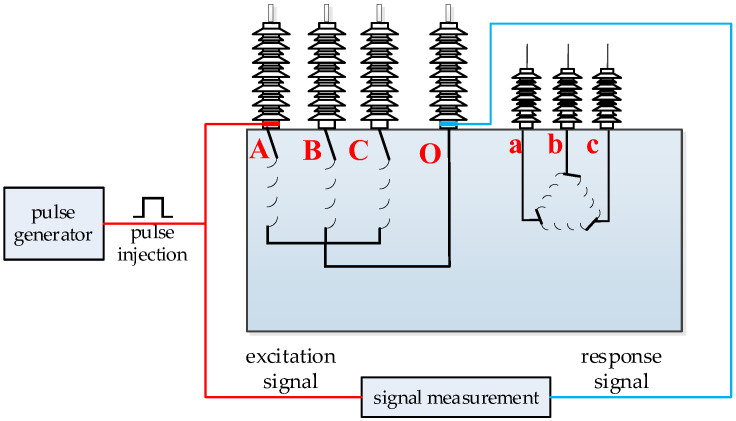
Schematic diagram of transformer experimental wiring (A, B, C, and O represent the high-voltage side phases and neutral point; a, b, and c represent the low-voltage side phases).

The test system wiring is configured as follows: the output terminal of the nanosecond pulse generator is coupled to the capacitive coupling sensor acting as the signal injection point. Simultaneously, the pulse generator output is linked to the first input channel of the signal acquisition device. The capacitive coupling sensor serving as the signal output point is then connected to the second input channel of the signal acquisition device. Finally, the sensors are mounted onto the corresponding bushing taps. The schematic diagram of the transformer experimental wiring is illustrated in Figure 12.

For star-connected windings, testing is conducted phase by phase, as exemplified by the high-voltage winding in the schematic. When testing the Phase A winding, the capacitive coupling sensor linked to the pulse generator is connected to the Phase A bushing as the excitation injection point, while the sensor connected to the signal acquisition device is installed on the neutral bushing as the response output point. Following parameter configuration, a nanosecond-level single pulse is triggered, concurrently activating the signal acquisition device to fully record the injection and response waveforms. Maintaining the output sensor configuration, the injection sensor is then safely transferred to the Phase B bushing to repeat the acquisition process. Finally, the injection point is relocated to the Phase C bushing to complete the phase testing.

For delta-connected windings, the test is carried out between phases, such as the low-voltage winding shown in the schematic diagram. Taking the test of Phase A low-voltage winding as an example, connect the capacitive coupling sensor linked to the pulse generator to the Phase A bushing as the excitation injection point, and install the capacitive coupling sensor connected to the signal acquisition device on the Phase B bushing as the response output point. Inject the pulse and collect the data. Afterwards, change the injection point of the pulse generator to Phase B and Phase C in sequence, and adjust the combination of the output-end sensors accordingly to complete the tests of Phase B and Phase C.

Following each acquisition, data files are immediately saved and labeled. Upon completion of all tests, data analysis is performed, frequency response curves are plotted, and these are compared against historical reference curves to evaluate the winding condition.

#### 3.2.2. Experimental Results and Analysis

##### 110 kV Transformer in Chongqing

To validate the efficacy of the method on field transformers, a decommissioned 110 kV power transformer in Chongqing was selected for impulse frequency response analysis (IFRA) based on pulse coupling injection. The experimental procedures were conducted under offline conditions, and three-phase frequency response curves for both the high-voltage (HV) and low-voltage (LV) windings were acquired. The primary nameplate specifications of the transformer are summarized in Table 1.

**Figure 13:**
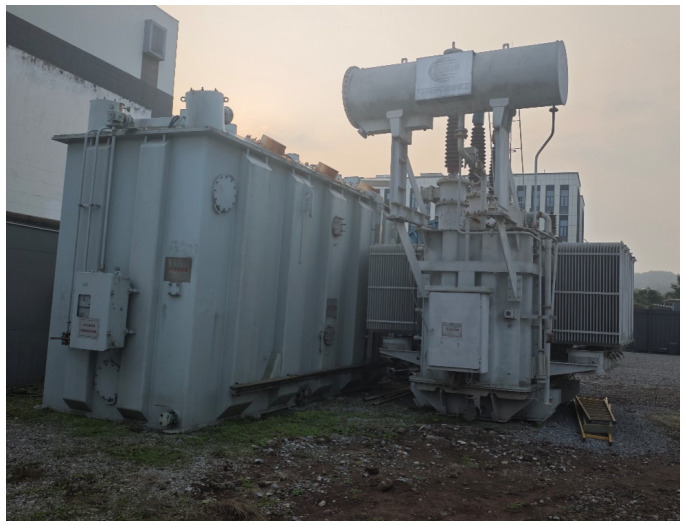
Field test photograph of the 110 kV transformer in Chongqing.

The high-voltage winding of the 110 kV power transformer is Y-connected, while the low-voltage winding is Δ-connected. During the test, excitation pulses with identical parameters were utilized for all experiments, specifically with an amplitude of 2000 V and a pulse width of 800 ns. The experimental setup is illustrated in Figure 13.

**Figure 14:**
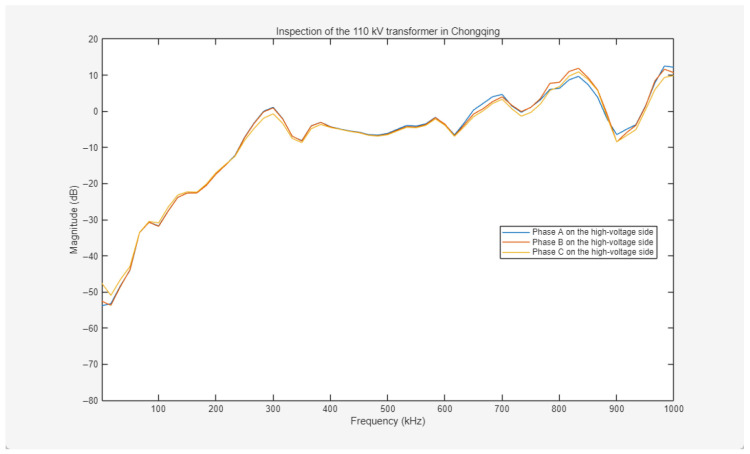
Frequency response curves of three-phase ABC on the high-voltage side of the 110 kV transformer in Chongqing.

The frequency response curves for the HV windings are presented in Figure 14. Within the 0–600 kHz low-to-mid frequency band, the response curves of Phase A and Phase B exhibit high coincidence. The amplitude deviation between Phase C and Phases A and B remains below 5 dB, representing a typical fluctuation within acceptable thresholds. In the 600–1000 kHz mid-to-high frequency range, the three-phase curves demonstrate a consistent variation trend, with amplitude differences maintained within 5 dB. The broadband curves exhibit typical healthy characteristics, including a steady amplitude rise at lower frequencies and a smooth trend at higher frequencies, without fault-indicative signatures such as sharp mutations, abnormal resonance peaks, or sudden amplitude drops. These results indicate that the distributed electrical parameters of the three-phase HV windings are consistent. Specifically, the inductances, reflecting the axial and radial structural integrity, and the capacitances, representing inter-turn and ground insulation characteristics, appear well-matched. No mechanical deformation, wire cake displacement, or insulation degradation is detected, with both electrical and mechanical performances remaining within healthy operating ranges.

**Figure 15:**
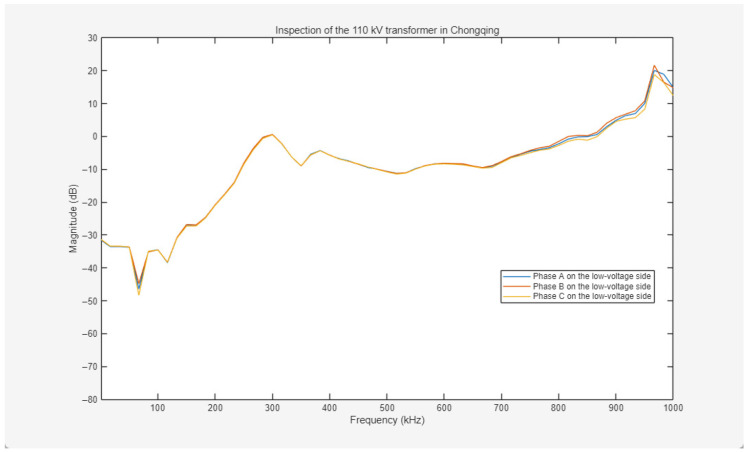
Frequency response curves of three-phase ABC on the low-voltage side of the 110 kV transformer in Chongqing.

The frequency response curves for the low-voltage (LV) windings are presented in Figure 15. Across the wide frequency range of 0–800 kHz, the three-phase responses of A, B, and C exhibit high coincidence. Within the higher frequency band of 800–1000 kHz, a minor amplitude deviation remains below 3 dB, while the three-phase variation trends remain highly synchronized without fault-indicative distortions or abrupt changes. Based on the fundamental physics of frequency response analysis, the low-frequency region primarily reflects the inductance characteristics and overall structural stability of the windings, whereas mid-to-high frequency bands correspond to insulation parameters such as inter-turn and ground capacitances. The smooth trajectories and minimal amplitude deviations of the LV side curves indicate excellent consistency in the distributed parameters of the windings. The slight high-frequency deviation is attributed to minute elastic deformations of the inter-cake supports or manufacturing and installation tolerances, rather than abnormal phenomena induced by winding deformation, insulation breakdown, or inter-turn short circuits.

Based on a comprehensive analysis of the frequency response curves on the high-voltage and low-voltage sides, combined with the features shown in the curves: high consistency of three-phase responses, amplitude differences within acceptable thresholds, and an absence of fault-indicative signatures across the full spectrum—no winding deformation, insulation degradation, or other electrical faults are detected in this 110 kV transformer.

##### 110 kV Transformer in Shenyang

IFRA based on pulse coupling injection was conducted on a newly manufactured 110 kV power transformer in Shenyang. The experimental procedures were performed under both online and offline conditions, and three-phase frequency response curves for both the high-voltage and low-voltage windings were acquired. The primary nameplate specifications of the transformer are summarized in Table 2.

**Figure 16:**
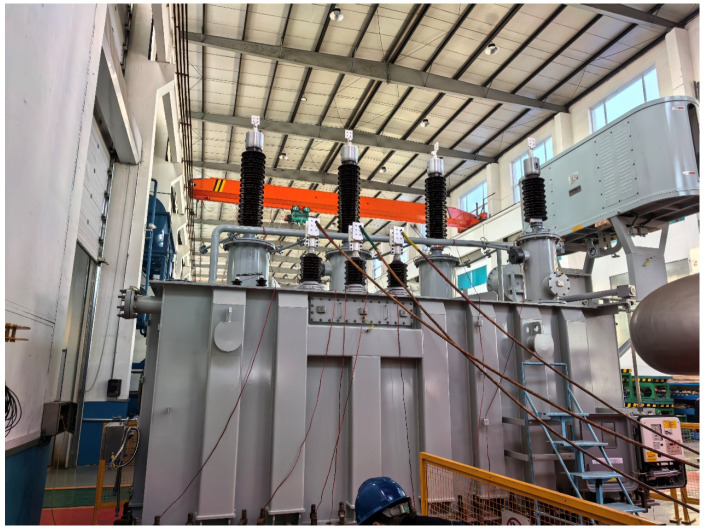
Field test photograph of the 110 kV transformer in Shenyang.

The high-voltage winding of the 110 kV power transformer is Y-connected, while the low-voltage winding is Δ-connected. During the testing, excitation pulses with identical parameters—specifically, an amplitude of 1000 V and a pulse width of 800 ns—were utilized for all experiments. The field test setup is illustrated in Figure 16.

**Figure 17:**
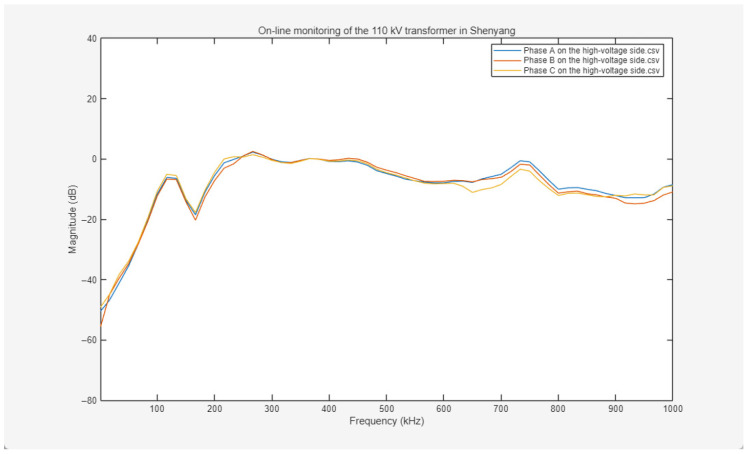
Frequency response curves of three-phase ABC on the high-voltage side measured on-line.

The online frequency response curves for the high-voltage windings are presented in Figure 17. These curves exhibit typical healthy characteristics across the full frequency range from 0 kHz to 1000 kHz. The variation trends among the three-phase responses (Phases A, B, and C) are highly consistent, with the waveforms being nearly coincident. Amplitude deviations across the low-, mid-, and high-frequency bands are consistently maintained within acceptable thresholds. The overall curves are smooth and continuous, lacking fault-indicative signatures associated with mechanical winding deformation, inter-turn short circuits, or insulation damage, such as sharp resonance peaks, drastic amplitude mutations, or discontinuous step-like drops. These results indicate that the high-voltage winding structure remains intact, with no significant geometric deformation or insulation degradation detected, while the electrical and mechanical characteristics of the three-phase windings maintain high consistency.

**Figure 18:**
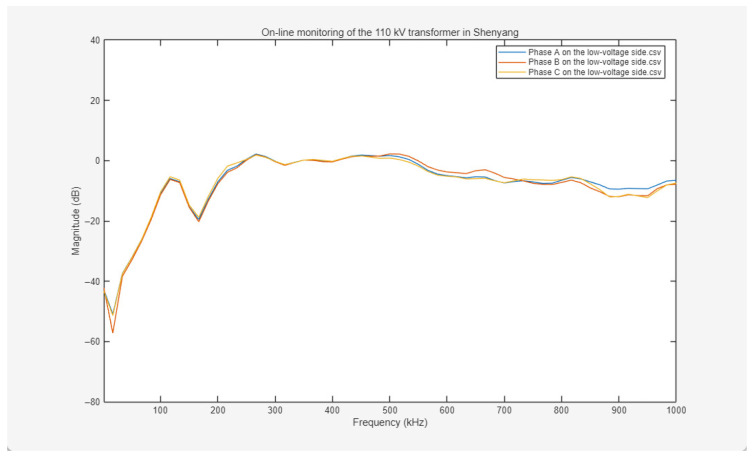
Frequency response curves of three-phase ABC on the low-voltage side measured on-line.

The online frequency response curves for the low-voltage windings are illustrated in Figure 18. The three-phase responses also maintain a highly synchronized variation trend across the 0–1000 kHz frequency range. The amplitude deviation is minimal and remains within the acceptable fluctuation range. The overall curves are smooth and continuous, with an absence of fault-indicative waveform distortions or abnormal amplitudes. Minor amplitude fluctuations in the low-frequency region are attributed to minute elastic deformations of inter-cake supports or manufacturing and installation tolerances, rather than abnormal phenomena induced by winding deformation, insulation breakdown, or inter-turn short circuits. This reflects that the electrical parameter alignment and mechanical structural stability of the low-voltage windings meet industrial standards.

Based on a comprehensive analysis of the online frequency response curves for both the high-voltage and low-voltage windings—characterized by high three-phase consistency, amplitude deviations within acceptable thresholds, and an absence of fault-indicative signatures—it is concluded that no winding deformation, insulation degradation, or other electrical faults are detected in this 110 kV transformer.

**Figure 19:**
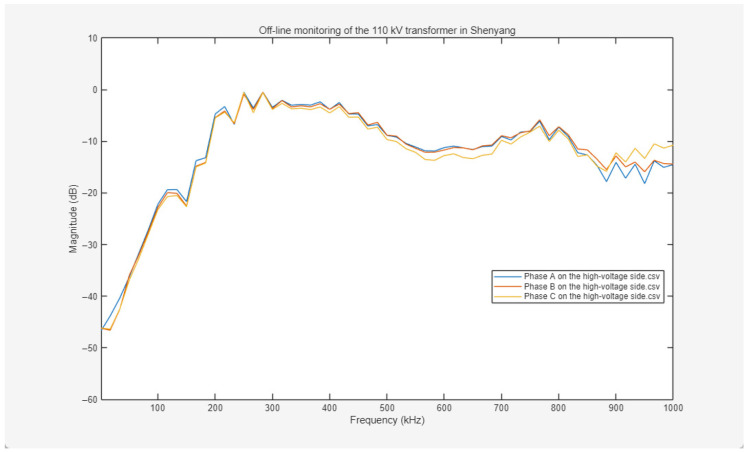
Frequency response curves of three-phase ABC on the high-voltage side measured off-line.

The offline frequency response curves for the high-voltage windings are presented in Figure 19. The responses of Phases A, B, and C exhibit a highly synchronized trend across the 0–1000 kHz frequency range, with amplitude deviations in each band remaining within acceptable thresholds. The overall curves are smooth and continuous, with only minor amplitude fluctuations in localized bands; notably, no fault-indicative signatures—such as sharp mutations, abnormal resonance peaks, or sudden amplitude drops—are observed. This indicates that the distributed parameters of the three-phase high-voltage windings are well-aligned, the axial and radial mechanical structures are stable, and no significant phenomena such as wire cake displacement or insulation aging are detected.

**Figure 20:**
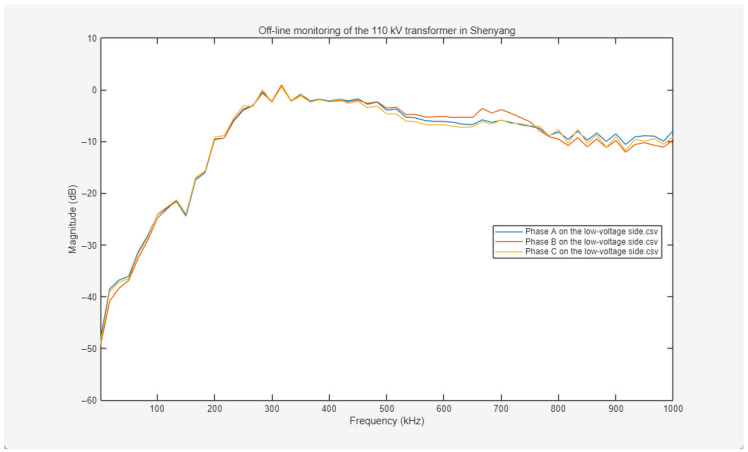
Frequency response curves of three-phase ABC on the low-voltage side measured off-line.

Offline frequency response curves for the low-voltage windings are presented in Figure 20. The three-phase responses (Phases A, B, and C) exhibit a highly consistent variation trend across the full frequency range. This consistency serves as a primary indicator for assessing the structural integrity of the windings, characterized by high synchronization across the scanned frequency spectrum. In the low-frequency region, which reflects the overall displacement and deformation of the windings, the curves exhibit close overlap; in the mid-frequency band, which is sensitive to internal inter-cake and inter-turn structures, the curves align in shape; and in the high-frequency region, which facilitates the detection of localized minor deformations, the curves maintain a consistent trend. Amplitude deviations remain minimal, falling within the acceptable dispersion bandwidth specified by power industry standards.

Further observation of the morphological evolution of the curves indicates that they follow the established characteristics of transformer winding frequency responses. In the initial low-frequency stage, the amplitude increases rapidly with frequency, a behavior primarily governed by the overall inductance of the windings. Within the mid-frequency band, the curves remain relatively stable, overlaid with limited-amplitude resonant peaks and valleys resulting from distributed LC networks within the windings. The entire response spectrum is continuous and lacks significant mutations or severe distortions.

Based on this comprehensive analysis—including the structural symmetry reflected by the high coincidence of the three-phase curves and the alignment of the spectral profiles with typical response laws—it is concluded that both the high-voltage and low-voltage windings of this Shenyang 110 kV transformer exhibit no significant fault indicators, and their mechanical integrity and electrical characteristics remain within satisfactory operational parameters.

##### 110 kV Transformer in Jiangjin, Chongqing

Impulse frequency response analysis (IFRA) based on pulse coupling injection was conducted on a 110 kV power transformer suspected of faults. The tests were performed under offline conditions, and three-phase frequency response curves for the high-voltage, medium-voltage, and low-voltage windings were acquired. Primary nameplate specifications are summarized in Table 3.

**Figure 21:**
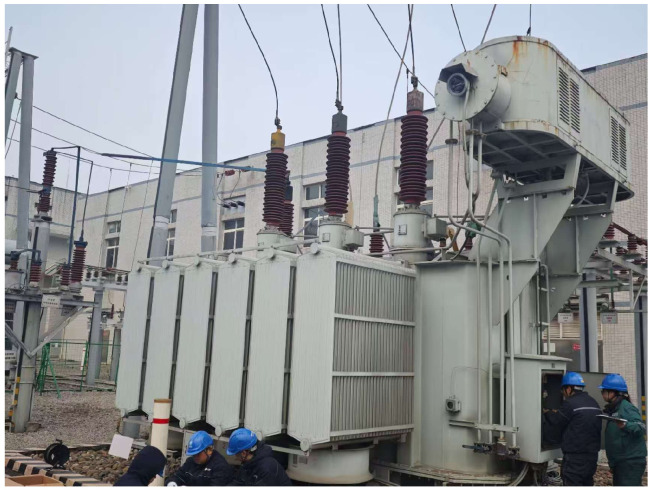
Field test photograph of the 110 kV transformer in Jiangjin, Chongqing.

The high-voltage and medium-voltage windings of the 110 kV power transformer are Y-connected, and the low-voltage winding is Δ-connected. During the testing, excitation pulses with consistent parameters—specifically, an amplitude of 1000 V and a pulse width of 800 ns—were utilized for the experimental series. The field test setup is illustrated in Figure 21.

**Figure 22:**
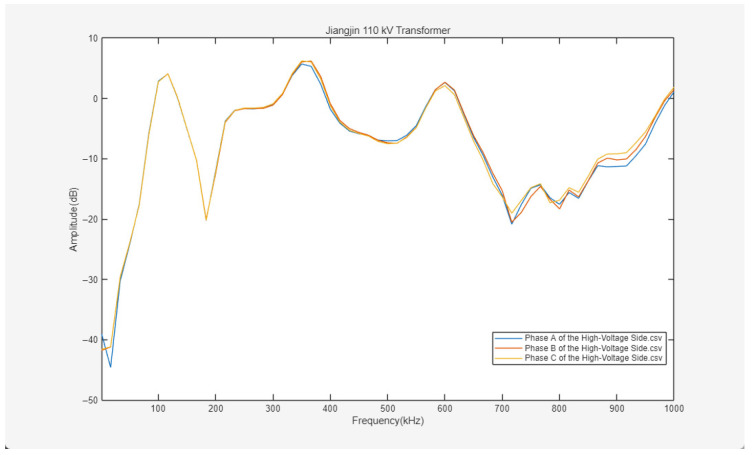
Frequency response curves of three-phase ABC on the high-voltage side.

**Figure 23:**
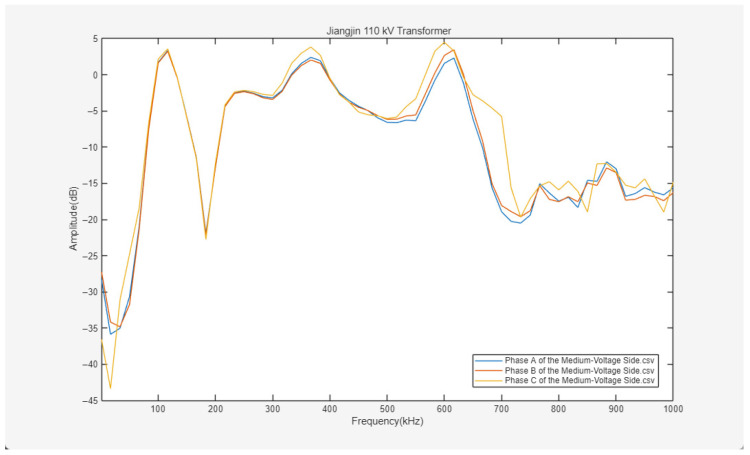
Frequency response curves of three-phase ABC on the medium-voltage side.

**Figure 24:**
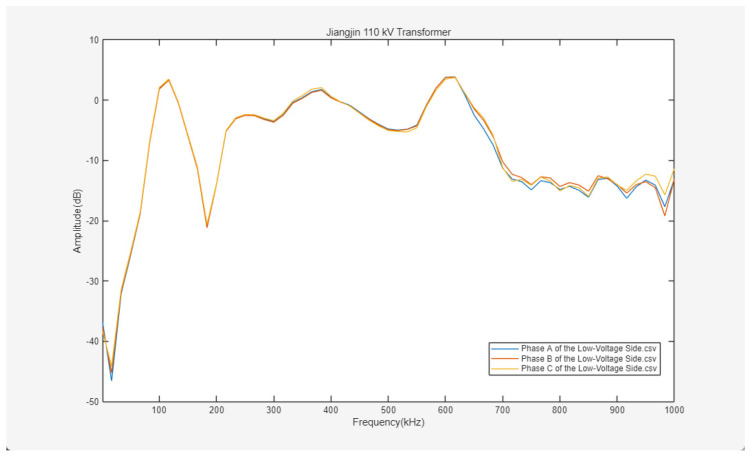
Frequency response curves of three-phase ABC on the low-voltage side.

The experimental results are shown in Figure 22, Figure 23 and Figure 24.

Figure 22, Figure 23 and Figure 24 present the frequency response curves for the high-voltage, medium-voltage, and low-voltage windings of the 110 kV transformer in Jiangjin, respectively. The horizontal axis of the spectra represents the frequency sweep range from 1 to 1000 kHz, while the vertical axis denotes the amplitude. Overall, the frequency response characteristics across different windings show a discernible differentiation trend, providing a clear basis for subsequent fault analysis.

As observed in Figure 22 and Figure 24, the three-phase frequency response curves of the high-voltage and low-voltage sides exhibit high coincidence across the broad sweep range. Such phase consistency serves as an indicator of the structural integrity and geometric symmetry of the windings, suggesting the absence of significant single-phase local defects such as inter-turn short circuits, winding deformation, or displacement. Furthermore, the curve profiles on both sides align with the established patterns of transformer winding frequency response. In the initial low-frequency stage, the amplitude increases rapidly with frequency, a behavior primarily governed by the overall inductance of the windings. Within the mid-frequency band, the curves remain relatively stable, overlaid with limited-amplitude resonant peaks and valleys resulting from distributed LC networks within the windings. The curves across the full frequency spectrum are continuous, without significant mutations or severe distortions, indicating that the mechanical integrity and electrical characteristics of the high-voltage and low-voltage windings remain in satisfactory condition.

In contrast, the frequency response of the medium-voltage winding illustrated in Figure 23 exhibits discernible abnormalities. While the Phase A and Phase B curves on this side exhibit high coincidence across the 1–1000 kHz range, the Phase C curve deviates from the others upon entering the mid-to-high frequency bands. This deviation manifests not only as differences in amplitude fluctuations but also as a shift in the overall trajectory of the curve. Such an anomaly in the mid-to-high frequency range is inconsistent with the typical response patterns observed on the high-voltage and low-voltage sides, suggesting that the medium-voltage Phase C winding may possess minor mechanical structural defects or electrical anomalies, such as localized wire cake displacement or inter-turn insulation degradation.

Based on this comprehensive analysis, the three-phase curves of the high-voltage and low-voltage windings of the Jiangjin 110 kV transformer exhibit high coincidence, reflecting structural symmetry and stable electrical characteristics without significant fault indicators. However, the frequency response of the medium-voltage Phase C in the mid-to-high frequency band differs from the other two phases, suggesting a potential minor fault in that specific winding. Further targeted detection is recommended to clarify the nature and location of this anomaly.

Due to the high cost and logistical complexity of internal inspections for 110 kV transformers, the reliability of the live monitoring results was verified through cross-validation with standardized offline tests. Prior to the pulse injection experiments, an offline SFRA test was conducted following the DL/T 911-2016 standard [23], which indicated the presence of C-phase deformation. The correlation between the live monitoring diagnostic conclusions and the offline benchmarks substantiates the reliability of the proposed method in detecting actual faults.

## 4. Winding Deformation Fault Diagnosis Algorithm

The intelligent diagnosis algorithm proposed in this section constitutes the software layer of the integrated framework. Its performance relies on the hardware infrastructure detailed in Section 2 and Section 3. High-sensitivity pulse signals captured by capacitive sensors serve as the primary input for the MSCNN–Transformer–PGA model. This closed-loop configuration facilitates the mapping of physical signal characteristics extracted by the hardware to corresponding fault types within the software architecture.

The case studies and experimental data acquired in Section 3 provide the empirical foundation for the intelligent diagnosis discussed herein. To effectively process complex fault signals and address the limitations of manual feature extraction, the MSCNN–Transformer–PGA model is proposed. The performance evaluation utilizes a hybrid dataset synthesized from the preceding experimental and simulation results. Specifically, the high-frequency pulse response signals extracted during the live 110 kV transformer field tests (Section 3.2) incorporate real-world background noise and physical sensor characteristics, enhancing the model’s practical credibility. Concurrently, augmented data from the verified simulations (Section 3.1) provide a comprehensive spectrum of fault types and severities. This approach addresses the scarcity of field fault samples, thereby mitigating overfitting and improving the model’s generalizability. Derived from the synergy of physical experiments and theoretical models, a total of 1200 valid samples were constructed, effectively bridging the hardware perception and software decision-making layers.

### 4.1. Winding Deformation Fault Diagnosis Based on MSCNN–Transformer–PGA Model

The MSCNN–Transformer–PGA model proposed in this study integrates the multi-band spatial feature extraction of a Multi-Scale Convolutional Neural Network (MSCNN), the global sequence modeling of a Transformer Encoder, and the dynamic feature focusing of a Physics-Guided Attention (PGA) mechanism. By incorporating prior electromagnetic physical knowledge of transformers, this architecture is designed to address the complex data processing demands of winding deformation diagnosis, enhancing both the precision and robustness of fault identification.

#### 4.1.1. Basic Principle

The MSCNN represents an advanced convolutional architecture designed for processing complex signals and time series. Unlike traditional networks that utilize a single fixed-size convolutional kernel, MSCNN simultaneously captures local features across various frequency bands by deploying parallel one-dimensional convolutional kernels of different scales. Its core mechanism leverages the parallel computing capabilities of multi-scale convolutional layers. When processing impulse frequency response data, this architecture can effectively capture both the fine-grained "burrs" and sharp resonance peak variations in high-frequency regions, as well as the broad, gentle fluctuations characteristic of lower frequencies. This multi-faceted feature extraction significantly accelerates the model’s training and inference processes.

The Transformer Encoder is a sequence modeling network primarily based on the attention mechanism, eschewing the recursive structures found in traditional recurrent neural networks. Through the Multi-Head Self-Attention mechanism, the Transformer directly computes correlations between any two positions in a sequence in parallel, allowing for a more comprehensive capture of global dependencies. Integrating the Transformer into the hybrid model effectively mitigates the gradient vanishing problem inherent in long-sequence processing. Furthermore, it facilitates the mining of deep-seated dependency information within the sequence data, providing robust decision support for identifying winding deformation faults.

The PGA mechanism is an algorithm designed to incorporate the diagnostic heuristics of human experts. By assigning varying weights to features across different frequency bands or time intervals, PGA enables the model to focus adaptively on critical information while dynamically adjusting its attention toward different input modalities. By introducing the physical failure mechanisms of transformer windings as prior constraints, PGA calculates physical attention scores and weights. This approach effectively filters irrelevant noise, enhances the model’s focus on fault-relevant features, and consequently improves diagnostic precision.

#### 4.1.2. Model Construction and Training

**Figure 25:**
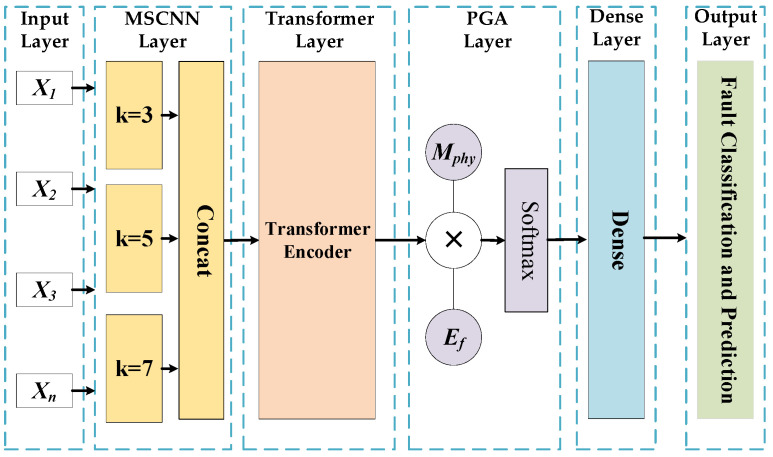
Structure diagram of the MSCNN–Transformer–PGA model.

This study introduces the deep learning model MSCNN–Transformer–PGA integrated with physical prior knowledge. Its primary framework is structured around a cohesive pipeline that facilitates the transition from multi-scale feature extraction and global sequence perception to physics-guided decision-making. The system architecture is illustrated in Figure 25.

Leveraging the MSCNN, Transformer, and attention mechanisms, this model incorporates physical prior knowledge to handle data under physical constraints through a three-tier processing architecture. Its global mathematical framework is formalized in Equation (4).

(4) $$Y=f_{PGA}(f_{Transformer}(f_{MSCNN}(X)))$$

During the initial feature extraction stage, the MSCNN module utilizes three parallel convolutional branches. For an input sequence *X*, the multi-scale local feature extraction process is defined in Equation (5).

(5) $$H_{k}=\sigma (W_{k}*X+B_{k}),\;\;k\in \{3,5,7\}$$

where *W_k_* and *B_k_* denote the weight matrix and bias vector of the convolution kernel of size *k*, respectively, and *σ* represents the activation function. The feature maps extracted across multiple scales are concatenated and subsequently integrated into the Transformer encoder.

Upon entering the Transformer module, positional encoding is applied to preserve temporal information, followed by the adoption of a multi-head self-attention mechanism to extract global contextual correlations. The computation of single-head attention is defined in Equation (6).

(6) $$\mathrm{Attention}(Q,K,V)=\mathrm{softmax}\left(\frac{QK^{T}}{\sqrt{d_{k}}}\right)V$$

where *Q*, *K*, and *V* denote the query, key, and value matrices, respectively, derived through linear transformations of the input features, and *d_k_* represents the scaling factor corresponding to the feature dimension. The multi-head mechanism enables the model to simultaneously attend to information from different positions across various representation subspaces.

In the feature fusion and output stage, the PGA module incorporates frequency-domain energy and the physical fault sensitivity matrix as modulation signals for the attention mechanism; its computation is defined by Equation (7).

(7) $$A_{phy}=\mathrm{softmax}(M_{phy}⊙E_{f}\cdot H_{trans})$$

where *M_phy_* is the pre-defined fault-feature correlation matrix based on physical prior knowledge, and *E_f_* represents the frequency-domain energy weight. This design enables the model to adaptively focus on fault-relevant information: the weights of features with high physical correlation are significantly enhanced, while those of low-correlation components or pure noise are effectively suppressed to minimize interference.

The transformer winding deformation fault dataset constructed in this study was acquired via an experimental platform, incorporating measured data for three typical faults: inter-cake spacing variation, radial deformation, and axial deformation. During the training process, the learning rate scheduling and dynamic batch size adjustments serve as two critical optimization methods, synergistically improving training efficiency and model performance. The learning rate utilizes a cosine annealing scheduling strategy with an initial value of 3 × 10^−4^ and a minimum value of 1 × 10^−6^. The core objective of this strategy is to facilitate rapid convergence in the early training stages and perform fine-grained optimization in later stages through a periodically varying learning rate. The batch size is fixed at 64, balancing training efficiency with numerical stability

Throughout the training process, the accuracy and loss curves for the training and validation sets remain consistent, with a deviation of less than 2%, indicating that no significant overfitting occurs during the training process. The model’s validation accuracy exceeds 95%. The training loss eventually stabilizes at approximately 0.08, while the validation loss stabilizes at approximately 0.18. This convergence behavior demonstrates that the training process is both stable and effective.

#### 4.1.3. Comparative Analysis of Model Performance

Following the training and validation of the MSCNN–Transformer–PGA model, ablation experiments were conducted to evaluate the effectiveness of the physics-guided mechanism. These experiments compare the performance of various model configurations within the context of transformer fault diagnosis, analyzing the influence of each module on multimodal feature fusion and providing a framework for model optimization.

As illustrated in Table 4, the MSCNN–Transformer–PGA model exhibits the highest overall performance. For the MSCNN–Transformer configuration lacking PGA physics guidance, the accuracy decreases to 94.2% due to the absence of physical mechanism-based screening. The Transformer–PGA model, which excludes multi-scale feature extraction, reaches an accuracy of only 90.8%, underscoring the critical role of multi-scale convolution in processing complex frequency response data. The performance of the complete model is attributed to the effective extraction of local features by the MSCNN, the Transformer’s capacity to capture global dependencies, and the enhanced robustness provided by the PGA’s dynamic weight adjustment.

Following the ablation experiments that substantiated the contribution of each module to model performance, a comparative analysis with traditional standalone models provides a broader evaluation of the MSCNN–Transformer–PGA model. Table 5 presents the fault classification accuracies for various traditional models using the same dataset.

Specifically, the accuracies of the SVM, MLP, and KNN models are 85.7%, 89.4%, and 83.9%, respectively. These results highlight performance variations when processing multimodal time-series data. The efficacy of the SVM model is sensitive to the selection of kernel functions and parameter tuning; given the complexity and high dimensionality of fault signals, identifying optimal configurations remains challenging. The MLP architecture is susceptible to overfitting, which may constrain its generalization on unseen test data. Furthermore, the KNN algorithm typically assumes uniform importance across all features, lacking an inherent mechanism for adaptive weight assignment. While traditional models encounter specific limitations in multimodal fault diagnosis, the proposed deep hybrid architecture addresses these challenges by integrating multi-scale feature extraction and physical constraints.

### 4.2. Fault Diagnosis Performance Analysis

In the field of transformer winding fault diagnosis, the IFRA method has emerged as a predominant and highly effective approach due to its acute sensitivity to subtle mechanical deformations. Such deformations directly alter the distributed inductance and capacitance parameters within the equivalent circuit, resulting in frequency shifts or pronounced amplitude variations in resonant peaks and valleys within specific frequency regions of the IFRA curve. However, IFRA signals span a wide frequency spectrum with high dimensionality and are susceptible to broadband electromagnetic interference during in situ live monitoring. Traditional single-feature extraction methods often struggle to simultaneously account for both local sensitivities and global trends.

The MSCNN–Transformer–PGA model proposed in this study is specifically optimized for the complexity inherent in individual IFRA signals. By utilizing the multi-scale convolutional kernels of the MSCNN, the model concurrently captures fine-grained "burr" variations in high-frequency regions and broad fluctuations in low-frequency regions. The Transformer module establishes the global dependencies of the broadband sequence. More importantly, the Physical-Guided Attention (PGA) mechanism incorporates the fundamental physical principles of transformer frequency response—such as the low-frequency region’s sensitivity to bulk structural integrity and axial displacement, and the mid-to-high frequency regions’ responsiveness to localized inter-cake capacitances and radial deformations—as prior constraints. This dynamically guides the model to focus on critical fault-indicative frequency bands.

To validate the diagnostic performance of the model when relying solely on impulse frequency response data, IFRA features from discrete frequency bands were extracted for independent testing according to physical regional divisions and compared against the model’s performance using broadband data. The results are summarized in Table 6.

The experimental results demonstrate that relying solely on single-band IFRA data, while indicative of certain fault types, presents discernible limitations. Low-frequency features often exhibit insufficient sensitivity in capturing incipient localized faults. Conversely, while mid-to-high frequency features are more sensitive, they are susceptible to misidentification due to frequency shifts induced by complex electromagnetic interference.

The multi-scale feature extraction and analysis of broadband IFRA data via the MSCNN–Transformer–PGA model addresses the limitations of traditional single-band analysis. Under the dynamic regulation of the PGA, the model adaptively assigns weights between low-frequency trends and high-frequency resonance points according to the physical evolution of various fault types. For instance, when identifying inter-cake short-circuit faults, the model automatically increases the feature weights within the 100–500 kHz range. Under simulated conditions with significant background noise, the PGA mechanism appropriately attenuates the weights of noise-dominated ultra-high-frequency components based on physical laws.

In summary, this deep learning strategy—integrating physical mechanisms, multi-scale feature extraction, and adaptive broadband attention—enables the model to capture critical distortion features reflecting the fault essence, ultimately achieving a diagnostic accuracy of 97.6%. This substantiates the feature analysis capabilities of the MSCNN–Transformer–PGA algorithm for complex frequency response signals and validates the robustness of this single-modal intelligent diagnosis method for engineering applications.

## 5. Conclusions

To address the challenges in traditional offline transformer winding deformation detection—such as the requirement for power outages, the low signal-to-noise ratio (SNR) of existing online monitoring technologies, and the limited feature extraction capabilities of diagnostic models—this study proposes an online monitoring technology and intelligent diagnosis method based on impulse coupling injection. Through collaborative innovation across detection hardware development, physical mechanism simulation, and deep learning algorithms, this approach overcomes the technical bottlenecks of high-precision online monitoring under non-outage conditions, providing robust technical support for the condition-based maintenance of power equipment.

Regarding hardware and measurement methods, this study introduces a custom-designed semi-ring capacitive coupling sensor tailored for high-voltage bushings. Utilizing a non-intrusive modular design, the sensor facilitates controllable high-frequency pulse injection and high signal-to-noise ratio response acquisition without requiring outages or modifications to original wiring. This design significantly reduces partial discharge risks and the costs associated with field installation and maintenance. Combined with a distributed parameter equivalent circuit model, this study conducts an in-depth simulation of the influence of typical faults—including inter-cake short circuits, inter-cake spacing variations, and radial deformation—on specific IFRA frequency bands, revealing the inherent physical correlations between fault evolution and frequency response distortion. Furthermore, offline and live field tests were conducted on multiple 110 kV transformers. While internal physical inspections were constrained by high engineering costs, the diagnostic reliability was rigorously validated through cross-validation against industry-standard offline SFRA benchmarks. The consistent performance observed across both healthy and defective units demonstrates that the proposed hardware–software collaborative approach is reliable and well-suited for practical engineering applications in complex environments.

In terms of intelligent fault diagnosis, this study proposes the MSCNN–Transformer–PGA deep hybrid model for the in-depth analysis of complex high-dimensional IFRA signals. The model utilizes an MSCNN to extract high-frequency local details and low-frequency trends in parallel, while establishing global dependencies across the full spectrum through a Transformer encoder. The innovatively introduced Physical-Guided Attention (PGA) incorporates the physical laws of transformer frequency response as prior constraints, dynamically guiding the model to adaptively focus on key frequency bands reflecting specific fault types while suppressing irrelevant noise.

Comprehensive performance evaluation shows that relying solely on impulse frequency response data, the MSCNN–Transformer–PGA model overcomes the limitations of traditional local frequency band analysis and achieves a fault classification accuracy of 97.6%. Compared with traditional machine learning algorithms, this deep model demonstrates significant advantages in feature capture and robustness when processing multimodal time-series data. This research provides an efficient, safe, and intelligent online monitoring solution for transformer winding deformation, contributing to the development of more reliable modern intelligent power systems.

## Figures and Tables

**Table 1:** Nameplate parameters of the 110 kV transformer in Chongqing.

Nameplate	Data
Type	SFZ9-40000/110
Rated Voltage (kV)	110 ± 8 × 1.25%/10.5
Rated Capacity (kVA)	40,000
Cooling Mode	ONAN/ONAF
Frequency (Hz)	50
Connection Group	Ynd11

**Table 2:** Nameplate parameters of the 110 kV transformer in Shenyang.

Nameplate	Data
Type	SZ20-50000/110
Rated Voltage (kV)	110/10.5
Rated Capacity (kVA)	50,000
Cooling Mode	ONAN
Frequency (Hz)	50
Connection Group	YNd11

**Table 3:** Nameplate parameters of the 110 kV transformer.

Nameplate	Data
Type	SFSZ9-40000/110
Rated Voltage (kV)	110/38.5/10.5
Rated Capacity (kVA)	40,000/40,000/40,000
Cooling Mode	ONAN/ONAF
Frequency (Hz)	50
Connection Group	YN yn0 d11

**Table 4:** Performance comparison of different models after ablation experiments.

Model	MSCNN–Transformer	Transformer–PGA	MSCNN–PGA	MSCNN–Transformer–PGA
Accuracy	94.2%	90.8%	92.5%	97.6%

**Table 5:** Performance comparison of different models.

Model	SVM	MLP	KNN	MSCNN–Transformer–PGA
Accuracy	85.7%	89.4%	83.9%	97.6%

**Table 6:** Fault diagnosis performance with IFRA features input from different frequency bands.

Feature Frequency Band	Type of Physical Information Included	Accuracy	Diagnosis Characteristic Analysis
Low-frequency band (<100 kHz)	Main magnetic circuit and overall distributed inductance parameters	82.5%	Sensitive to severe axial deformation and overall displacement, but prone to missing minor local faults.
Medium-high frequency band (100 kHz–1 MHz)	Winding distributed capacitance and local structural characteristics	86.3%	High sensitivity to inter-cake short circuits and minor radial deformations, but susceptible to high-frequency noise interference in field tests.
Full-band deep features	Including global trend and local resonance details	97.6%	Full-band adaptive focusing guided by physical mechanism, achieving both high sensitivity and strong noise immunity.

## Data Availability

Dataset available upon request from the authors.
